# Effectiveness of the AS03-Adjuvanted Vaccine against Pandemic Influenza Virus A/(H1N1) 2009 – A Comparison of Two Methods; Germany, 2009/10

**DOI:** 10.1371/journal.pone.0019932

**Published:** 2011-07-18

**Authors:** Helmut Uphoff, Matthias an der Heiden, Brunhilde Schweiger, Hartmut Campe, Dietmar Beier, Carina Helmeke, Martina Littmann, Walter Haas, Silke Buda, Daniel Faensen, Marcel Feig, Doris Altmann, Ole Wichmann, Tim Eckmanns, Udo Buchholz

**Affiliations:** 1 Hessisches Landes Prüfungs - und Untersuchungsamt im Gesundheitswesen, Dillenburg, Germany; 2 Abteilung Epidemiologie für Infektiöse Erkrankungen, Robert Koch-Institut, Berlin, Germany; 3 Nationales Referenzzentrum für Influenza, Robert Koch-Institut, Berlin, Germany; 4 Bayerisches Landesamt für Gesundheit und Lebensmittelsicherheit, Oberschleißheim, Germany; 5 Landesuntersuchungsanstalt für das Gesundheits - und Veterinärwesen Sachsen, Dresden, Germany; 6 Fachbereich Hygiene, Landesamt für Verbraucherschutz, Magdeburg, Germany; 7 Abteilung Gesundheit, Landesamt für Gesundheit und Soziales, Rostock, Germany; University of Rochester School of Medicine, United States of America

## Abstract

During the autumn wave of the pandemic influenza virus A/(H1N1) 2009 (pIV) the German population was offered an AS03-adjuvanted vaccine. The authors compared results of two methods calculating the effectiveness of the vaccine (VE). The test-negative case-control method used data from virologic surveillance including influenza-positive and negative patients. An innovative case-series methodology explored data from all nationally reported laboratory-confirmed influenza cases. The proportion of reported cases occurring in vaccinees during an assumed unprotected phase after vaccination was compared with that occurring in vaccinees during their assumed protected phase. The test-negative case-control method included 1,749 pIV cases and 2,087 influenza test-negative individuals of whom 6 (0.3%) and 36 (1.7%), respectively, were vaccinated. The case series method included data from 73,280 cases. VE in the two methods was 79% (95% confidence interval (CI) = 35–93%; P = 0.007) and 87% (95% CI = 78–92%; P<0.001) for individuals less than 14 years of age and 70% (95% CI = −45%–94%, P = 0.13) and 74% (95% CI = 64–82%; P<0.001) for individuals above the age of 14. Both methods yielded similar VE in both age groups; and VE for the younger age group seemed to be higher.

## Introduction

Phase 6 of the 2009 pandemic caused by the influenza virus A/(H1N1) 2009 (pIV) started with the announcement of the World Health Organization on June 11, 2009. For countries of the Northern hemisphere, including Germany, a vaccine became available after the autumn wave had already started. In Germany for the most part of the autumn wave, only one type of vaccine was available which was an egg-based vaccine containing 3.75 µg hemagglutinine as antigenic component and the adjuvant AS03.

Prior to the pandemic it was anticipated that a pandemic vaccine - even when adjuvanted - needs to be given twice to induce protective immunity [1]. However, tests with the pandemic vaccine containing pIV antigen have shown that a single vaccine dose of 15 µg without adjuvant may be sufficient in participants between 3 and 77 years of age [2]. Other studies suggested that a single dose of squalen-adjuvanted vaccine directed against pIV may induce sufficient levels of immunity in adults (using 3.75 µg hemagglutinine) [3] and even in children 6–36 months old (using 1.9 µg antigen) [4].

In Germany, the vaccine adjuvanted with the squalene AS03 became available to the population from week 44/2009 onwards. The German standing committee for vaccination recommended the vaccine for the entire population, prioritizing medical personnel, persons with chronic underlying conditions and pregnant women [5]. While initially two doses were recommended for children up to 9 years and elderly persons, an updated statement recommended a single dose for all age groups [6].

Recently, Orenstein has compared several methods to estimate VE for influenza from observational data including the test-negative case-control method [7]. To extend the repertoire of observational study types incorporating the fact that the vaccination campaign occurred concurrently with the epidemic wave we attempted to explore another method which has not been described previously and uses nationally reported cases of influenza only. The method is motivated by the self-controlled case series method that has been used in studies on vaccine safety [8].

The objective of this paper is to assess VE of the AS03-adjuvanted pIV vaccine using two methods: first, the test-negative case control method; second, a novel form of a case-series method.

## Materials and Methods

### Laboratory test-negative case-control method

For this evaluation only patient samples taken with diagnostic intent were analysed. Due to German standards, ethics approval and informed consent was not necessary.

We used the test-negative case-control study design similar to that described in the ECDC technical document (2009) [9]. We obtained virologic surveillance data from the National Reference Center for Influenza at the Robert Koch-Institute (RKI), and the State Laboratories of Mecklenburg-Western-Pommerania (Rostock), Saxonia-Anhalt (Magdeburg), Bavaria (Oberschleissheim) and Saxonia (Dresden), Germany. Samples were provided by physicians who swabbed patients with influenza-like illness (ILI). Samples were accompanied by a patient-based questionnaire with information on age, sex, state of residence, date of symptom onset, symptoms, underlying disease (cardiovascular, respiratory, diabetes) and dates of vaccination against seasonal and pandemic influenza, if any. Cases were confirmed by reverse-transcriptase polymerase chain reaction (RT-PCR), controls were RT-PCR negative. Hospitalized ILI patients and test-negative patients where swabs were taken more than four days after disease onset were excluded. Because vaccination against pandemic influenza virus started in week 44/2009, we restricted data analysis to patients with illness onset between week 44/2009 and 07/2010. For seasonal and pandemic A/H1N1 2009 influenza vaccine it has been shown that 90% and 79% of vaccinees, respectively, had protective antibody titres two weeks after vaccination [10], [11]. We defined therefore patients as vaccinated when more than two weeks had passed after the date of vaccination, and as not vaccinated when less than two weeks had passed after the date of vaccination.

For the analysis of categorical variables we calculated odds ratios (OR) and 95% confidence intervals (CI). We built two models describing the effect of vaccination against pIV and other independent factors on pIV infection, one for children (less than 14 years) and one for “adults” (at least 14 years). We considered as confounders age (as numerical variable), sex, illness week and location of residence, and included in the models those variables that were associated with both pIV infection and vaccination and changed the OR for vaccination by more than 5%. The final model included only the remaining variables as well as illness week. Statistical tests were two-sided and *P* values of less than 0.05 were considered statistically significant. For calculations we used the software Stata (Stata Corporation, College Station, Texas, USA).

### Case-series method

For the case-series method we used all cases of pIV notified through the mandatory notification system where date of disease onset and age was known. Laboratory-confirmed cases of pIV had to be notified by the diagnosing laboratories to the public health system where vaccination status was investigated. Final data were reported via state health departments to the RKI. Hospitalized cases were not excluded, but were rare (less than 2%).

For this method we needed to define a time interval after influenza vaccination when an immune response is beginning to be detectable and when it is fully mounted. According to Brokstad et al. [12] hemagglutinine antibody titers started to increase after 8–9 days after vaccination. Day 7 would thus be the “last day” in the “unimmune” period and infection on day 7 results in illness on average on day 8 or 9 (adding 1.5–2 days of incubation period). As explained for the test-negative case-control method most vaccinees develop protective antibody titres 2 weeks after vaccination [10], [11]. We assumed therefore a period of lacking protection after vaccination until day 7 (i.e. day 9 when the day of illness onset is used) and a period of full protection from day 14 after vaccination.

To calculate the VE we assumed that the chance to fall ill and get notified is a proxy for the force of infection that affects all persons. So the cumulative force of infection that a person is exposed to during time t is represented by the number of cases reported during time t. We assume that the chance to be selected for notification is similar in all vaccinated persons during the protected and unprotected phase and we assume that the susceptibility of the people at the time of vaccination is similar to the susceptibility in the population. Susceptibility in the vaccinated group changes in relation to the unvaccinated group from the time of mounted immunity due to induced protection. We assumed further that persons who were immunized were not protected in the immediate time period after vaccination. Based on these assumptions the number of reported cases in a cohort defined by their vaccination date within a given week during any period is a result of 1. the number of individuals exposed, 2. their susceptibility, and 3. the cumulative risk to become ill and be notified during that period.

As the number of individuals in each weekly vaccinated cohort is unknown but unchanged over time, the ratio of (cumulative force of infection during the unprotected phase/number of cases in the weekly vaccinated cohort during this unprotected phase) to the (cumulative force of infection during the protected phase/number of cases from the cohort during the protected phase) indicates a change in susceptibility which is attributed to the effect of the vaccine.

To determine the force of infection during the unprotected period of a cohort vaccinated in a given week we assumed that on average people were vaccinated (and from thereon exposed to infected cases) after Wednesday. While we calculated the exact difference between vaccination date and illness date in days we used weekly data to calculate the force of infection. For an assumed 9 day unprotected period (to the day of symptom onset) the exposure to infection for a vaccine cohort of week x would be the number of all reported cases (vaccinated and unvaccinated) of week x multiplied with 4/7(exposure time in week x), plus the number of all reported cases of week (x+1) multiplied with 5/7 (exposure time in week (x+1)). For the number of cases vaccinated in week x that occurred within the respective unprotected period of 9 days we counted the number of cases that were vaccinated in week x and had a date of illness onset within 9 days after the date of vaccination. Thus, the reference value for a cohort of week x (term 1) was:

(all reported cases of week x *4/7 + all reported cases of the following week (x+1)* 5/7)/(number of cases during the unprotected period (illness onset within 9 days after vaccination) of the cohort vaccinated in week x)

We then performed the same calculation for the assumed protected period which yielded the number of cases necessary to generate a vaccinated case in the hypothetically protected period ending with week 53/2009. The calculation for the protected period beginning 14 days after vaccination for a weekly cohort would be (term 2):

(sum of all reported cases with illness onset between week (x+2) and week 53)/(number of cases during the protected period among those vaccinated in week x with illness onset between week (x+2) and week 53)

The ratio of both terms (term1/term2) gives the relative risk which – under the null hypothesis – is one if the vaccine had no effect.

For the calculation of the overall effect (pooled for all vaccinated weekly cohorts) we assumed that the relation between the force of infection and the generation of cases is stable over time and simply pooled the numerator and denominator data of the respective terms and weeks to calculate an average of the total VE (appendix).

Regarding the final data set used, we focused on the period from week 44 (when vaccination started) to week 53 when the epidemic virus circulation had largely ceased. Weeks where no vaccinated case was reported neither in the unprotected nor in the protected period, were excluded. We conducted the following two sensitivity analyses: (a) for the determination of the force of infection we included only cases with known age and illness onset; however, cases with unknown vaccination status were excluded or included; (b) the unprotected period was varied lasting for 6,7,8,9 or 11 days, respectively (keeping the assumed protected period constant beginning 15 days after vaccination).

The relative risks were calculated for each week of vaccination and for two age groups (less than 14 years, 14 years or above). We report relative risks for weekly cohorts of vaccination as well as an overall estimate for all persons vaccinated.

## Results

### Laboratory test-negative case-control method

**Figure 1:**
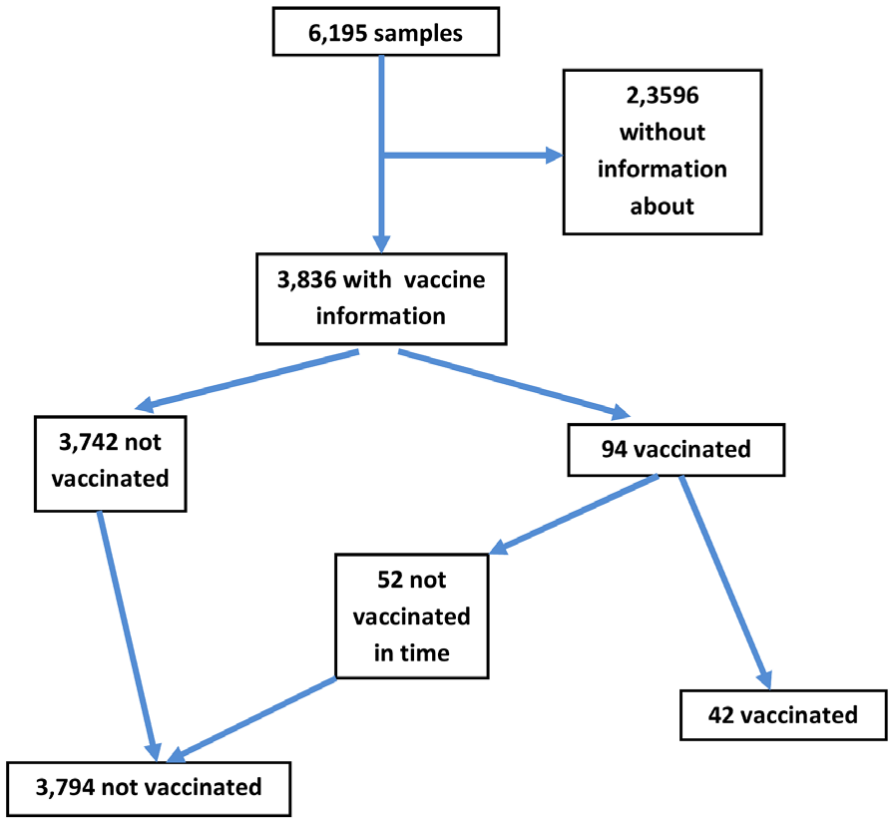
Breakdown of population according to vaccination status, test-negative case-control method.

There were 6,195 samples of patients with illness onset between week 44/2009 and 07/2010. Of these, 2,837 (46%) were positive for pIV (Tab. 1). Information on explanatory variables was available in 43% (underlying illness; minimum) up to 99% (age). Median age was 12 years (interquartile range: 6–26 years). Hundred twenty-six patients were reported to be vaccinated against pIV. Of these, information on vaccination date was available for 94 (75%). Among patients with vaccination date data, the proportion of positive samples (positivity rate) dropped with increasing interval between vaccination and disease onset. In 42 patients illness onset was later than 14 days after vaccination with the pandemic vaccine representing 1% of patients with information on vaccine status. Twenty-seven (64%) of the 42 patients were aged younger than 14 years of age and 15 (36%) were 14 years or older. For analysis of the VE, only patients could be included who were known to be unvaccinated or who were vaccinated and where date of vaccination was available. This was the case for 3,836 patients (Fig. 1). ILI cases with information about their vaccination status were more likely to be children compared to ILI cases without information about their vaccination status. Other variables, such as sex, positivity to pIV and chronic underlying disease were not significantly different between the two groups.

**Table 1:** Basic data used in the test-negative case-control study and the case-series study.

	Data from national reference and state laboratories (test-negative case-control method)					Data from reporting system (case-series method)	
Variable	With information		among those with information				
	N	%		N	%	N	%
Lab result for pIV	6195	100%	positive	2837	46%	NA	NA
			negative	3358	54%	NA	NA
Vaccination against pandemic A/(H1N1) 2009	3836	62%	Vaccinated more than 14 days before illness onset	42	1%	57	0.1%
			Not vaccinated or not in time	3794	99%	73229	99.9%
Vaccination against seasonal influenza	5180	90%	Vaccinated more than 14 days before illness onset	491	9%	NA	NA
			Not vaccinated or not in time	4689	91%	NA	NA
Age	6156	99%	0–9	2452	40%	18877	26%
			10–19	1797	29%	28634	39%
			20–29	568	9%	8173	11%
			30–39	437	7%	5671	8%
			40–49	459	7%	6963	10%
			50–59	300	5%	3715	5%
			60–69	77	1%	853	1.2%
			70+	66	1%	393	0.5%
Sex	6124	99%	Male	3173	52%	36628	50%
			Female	2951	48%	36387	50%
Underlying disease	2653	43%	Respiratory	237	9%	NA	NA
			Cardiovascular	75	3%	NA	NA
			Diabetes	28	1%	NA	NA
			unspecified	141	5%	NA	NA
			none	2172	82%	NA	NA

Completeness and frequency distribution of variables describing (a) the study population of patients whose sample was sent to the National Reference Center for Influenza or to one of four state laboratories and tested for pandemic influenza virus A/(H1N1) 2009 (test-negative case-control method); and (b) characteristics of the patients reported to the public health system (used in the case-series method); Germany, 2009/10.

Of 1,749 pIV cases six (0.3%) were vaccinated more than 14 days before illness onset, of 2,087 test-negative individuals 36 (1.7%) were vaccinated. The six vaccinated cases were all treated by different physicians. Four (67%) were younger than 14 years (9–13 years), and of these 2 had an underlying chronic condition; two (33%) were at least 14 years old (51 and 64 years), and of these, one had a chronic underlying condition.

**Figure 2:**
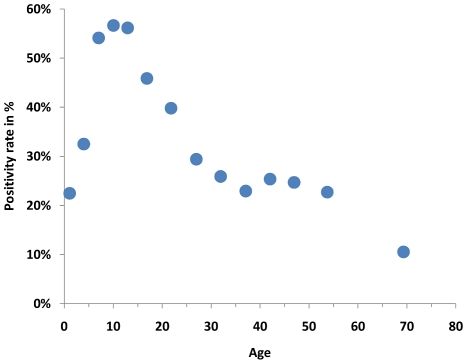
Proportion of positive samples (positivity rate) by age. Points are located at the mean of the ages in the age groups; Germany 2009/2010.

In **univariate** analysis, vaccination against pIV was associated with pIV infection with an OR of 0.17 (95% CI = 0.06–0.40; *P*<0.001), however, vaccination against seasonal influenza (OR = 0.91; 95% CI = 0.95–1.10; *P* = 0.34) and underlying disease (OR = 0.84; 95% CI = 0.69–1.04; *P* value = 0.10) were not. Positivity rate differed by age. It first increased up to 10 years of age and then declined thereafter (Fig. 2). Also male sex (OR = 1.14; 95% CI = 1.03–1.26; *P* = 0.01) was significantly associated with pIV infection. Positivity rate varied by week of illness onset with a plateau between week 44 up to week 52 and declined thereafter.

After m**ultivariate** logistic regression the model for children included the variables vaccination against pIV, age and illness week, and the model for adults contained the variables vaccination against pIV, age, illness week and state of residence (Tab.e 2). In children vaccination against pIV had an OR of 0.21 (Tab. 2) yielding an effectiveness of 79% (95% CI = 35%–93%; *P* = 0.007). In the age group 14 years and above, vaccination against pIV had an OR of 0.30 for an effectiveness of 70%, but this result was not statistically significant (*P* = 0.13; Tab. 2).

**Table 2:** Multivariate logistic regression model, test-negative case-control method.

	OR	Lower CL	Upper CL	p-value
Below 14 years				
Vaccination against pIV	0.21	0.07	0.65	0.007
Age less than 11 years; per year	1.21	1.18	1.26	<0.001
Age more than 10 years; per year	0.84	0.74	0.96	0.009
At least 14 years				
Vaccination against pIV	0,30	0.06	1.45	0.13
Age 14 years and above; per year	0.97	0.96	0.98	<0.001

Final multivariate logistic regression model of explanatory variables for infection with pandemic influenza (pIV) controlling for illness week (below 14 years) and for illness week and state of residence (at least 14 years); laboratory test-negative case-control method; Germany, 2009/10.

### Case-series method

**Figure 3:**
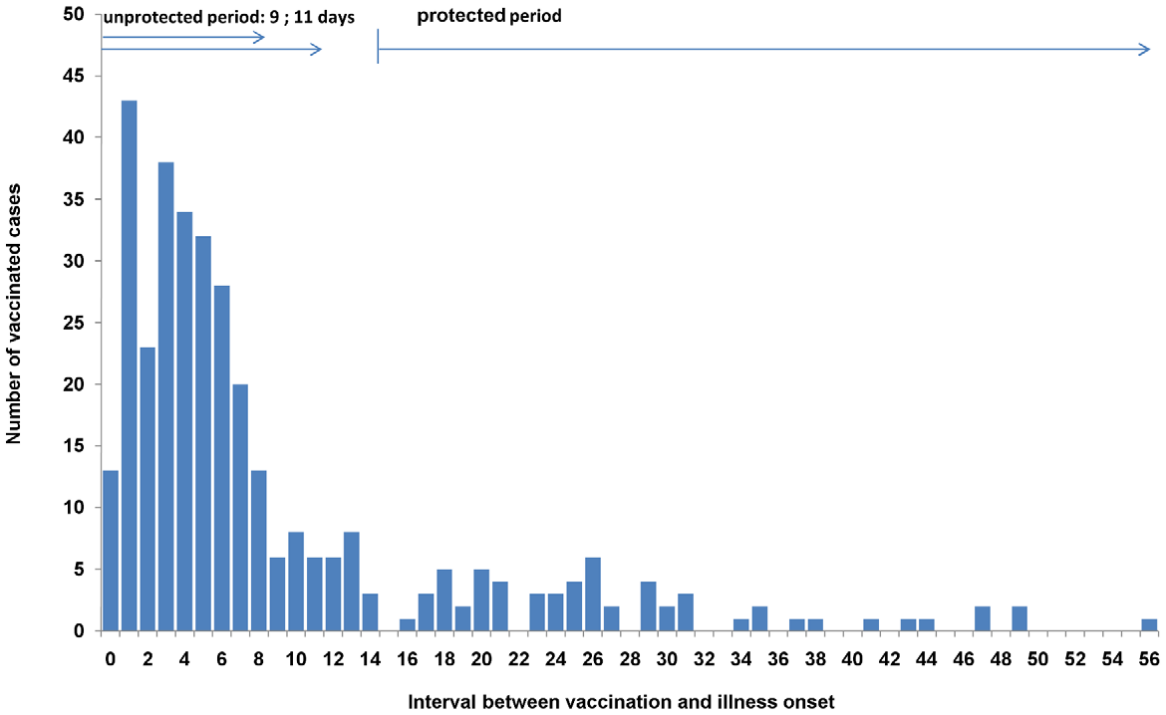
Vaccinated cases by time between vaccination and Illness. Frequency of reported and vaccinated laboratory-confirmed cases of pandemic influenza H1N1(2009) by interval between vaccination and illness onset; weeks of illness onset 44–53/2009; Germany.

**Figure 4:**
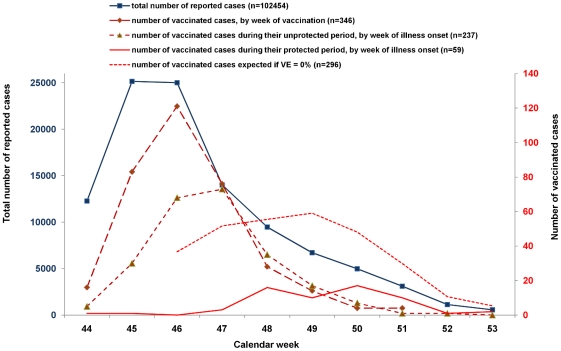
Case-series method. Frequency of total number of cases with known age and onset of illness (grey, left y-axis), of vaccinated cases by week of vaccination (dashed grey line; right y-axis), vaccinated cases by week of illness onset (unprotected period; dashed-dotted line; right y-axis), vaccinated cases by week of illness onset (protected period; black line; right y-axis) and vaccinated cases by week of illness onset that would be expected if the vaccine had no effect (protected period; dashed black line; right y-axis), Germany; week 44–53, 2009.

In the period from week 44/2009 to week 53/2009 102,454 cases with a known date of illness onset and age were reported. Of these, vaccination status was known for 73,280 cases (71%). The number of vaccinated cases by interval between vaccination date and illness onset started with a peak on the first day after vaccination and decreased then rapidly to very low numbers by day 10–14 (Fig. 3). The distribution of the number of vaccinated cases by age group was bimodal with one peak at the age group 11–15 years and a second at 46–50 years. Figure 4 shows the total number of cases by week of illness onset as well as the number of vaccinated cases by week of vaccination. The shape of the curve of vaccinated cases by week of vaccination is similar to that of the number of reported cases. The expected number of vaccinated cases during their protected period (assuming that VE is 0%) differs markedly from the curve with the actual number of vaccinated cases during the protected period (Fig. 4).

**Figure 5:**
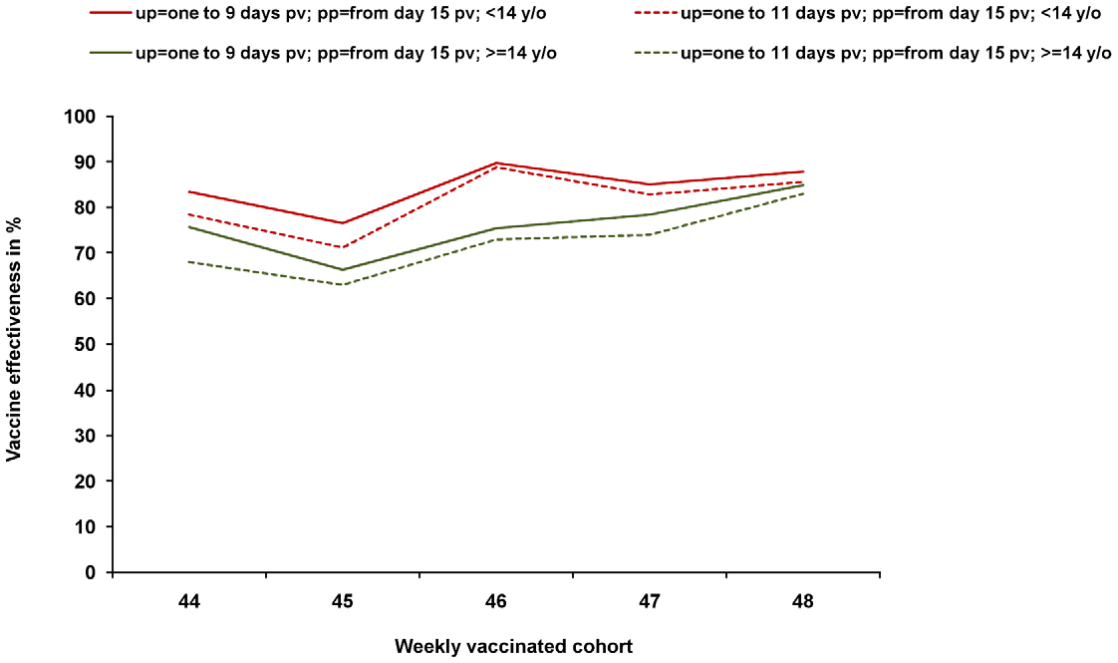
Vaccine effectiveness by weekly cohort. Vaccine effectiveness of pandemic AS03-adjuvanted vaccine, by weekly vaccinated cohort; case-series method; force of infection is represented by all reported cases with available information on age, illness onset and vaccination status; for methods: see text; up = unprotected period, pp = protected period, pv = post vaccination.

The VE estimates for the different weekly cohorts is relatively stable within the two age segments despite the low numbers of vaccinated cases (Tab. 3; Fig. 5). In the sensitivity analysis, neither choosing different lengths of the unprotected period (6,7,8,9 and 11 days, respectively) nor the inclusion or exclusion of cases where the exact vaccination status is unknown (for the parameter “total number of reported cases”) has a marked effect on the estimated VE. The confidence intervals of the weekly cohorts overlap. The main influence seems to be age. Overall VE is higher in children compared to adults. The range of the overall point estimates is 86–89% in children and 69–75% in adults, for the weekly cohorts it ranges from 71–90% in children and from 58–85% in adults. All point estimates are statistically significant. Excluding people over the age of 60 from analysis in the case-series method raises the VE estimate by 5% for the age group 14–60 years.

**Table 3:** Vaccine effectiveness by week of vaccination calculated by the case-series method.

			VE by weekly vaccinated cohort						Total number of reported cases (up)	Vaccinated cases (up)	Total number of reported cases (pp)	Vaccinated cases (pp)	VE	Lower 95% CL	Upper 95% CL
		Age group(years)	Calendar weeks												
Unprotected period	Protected period		44	45	46	47	48	49							
**Force of infection represented by all reported cases with information on date of illness onset and age**															
one to 9 days pv	from day 15 day pv	all ages	74	67	80	81	86	85	119001	236	163770	62	81	75	86
one to 9 days pv	from day 15 day pv	<14	81	74	89	84	88	-	49503	107	71814	19	88	80	92
one to 9 days pv	from day 15 day pv	> = 14	72	62	71	78	85	80	63766	125	89448	43	75	66	83
one to 11 days pv	from day 15 day pv	all ages	67	62	78	78	84	83	144280	249	163770	62	78	71	83
one to 11 days pv	from day 15 day pv	<14	76	68	88	82	85	-	60207	111	71814	19	86	77	91
one to 11 days pv	from day 15 day pv	> = 14	64	58	68	74	83	79	77280	133	89448	43	72	61	80
**Force of infection represented only by reported cases with information on date of illness onset, age and vaccination status**															
one to 9 days pv	from day 15 day pv	all ages	78	71	83	82	86	84	85449	235	126494	61	82	77	87
one to 9 days pv	from day 15 day pv	<14	83	77	90	85	88	-	34929	107	54877	19	89	82	93
one to 9 days pv	from day 15 day pv	> = 14	76	66	75	78	85	79	45974	124	69638	42	69	53	79
one to 11 days pv	from day 15 day pv	all ages	71	66	81	79	84	83	103804	248	126494	61	80	73	85
one to 11 days pv	from day 15 day pv	<14	78	71	89	83	86	-	42590	111	54877	19	87	78	92
one to 11 days pv	from day 15 day pv	> = 14	68	63	73	74	83	78	55828	132	69638	42	74	64	82

Results are given for different lengths of the unprotected period (9 vs 11 days, respectively; first column); for all ages, those aged less than 14 years and 14 years and above (third column); and when the number of total cases exercising force of infection excludes (lower half) or includes (upper half) reported cases with unknown vaccination status. Cells are empty for weeks where no vaccinated case was reported neither in the unprotected nor in the protected period; pv = post vaccination; up = unprotected period; pp = protected period; VE = vaccine effectiveness; CL = confidence limit.

## Discussion

Based on data from virologic surveillance as well as from the notifiable disease surveillance system we have found evidence for a good clinical protective effect of the AS03-adjuvanted vaccine that was used in Germany against pIV in autumn of 2009. This finding is corroborated by three results: (1) The positivity rate of vaccinated persons decreased with increasing interval between vaccination date and illness date; (2) the number of vaccinated cases in the database of reported cases decreased to low numbers for those who were vaccinated more than 10–14 days before illness onset (Fig. 2); (3) using two different data sources and two different statistical methods have led to similar point estimates of effectiveness in two age strata.

The dataset used for the test-negative case-control method comprises detailed data from sentinel physicians, but the number of vaccinated cases was small. The dataset from the mandatory notification system - used for the case-series method - comprised in principle data from all physicians and hospitals who diagnose pIV, is many-fold larger but includes fewer patient parameters. Due to these differences, not all stratifications or analyses were similarly possible for both methods. In both datasets persons over 60 years were rare; excluding them in the case-series method would have led to an increase of 5% in the 14–60 year old age group indicating a relatively lower protection in the elderly.

The test-negative case-control method was first described by Skowronski [13] and Uphoff [14]. It provides a convenient set of controls and can control for a number of covariates if collected. It is based on the assumption that vaccinated patients consult their physician with the same likelihood as non-vaccinated patients when developing ILI and that influenza is detected with the same likelihood if present [13]. To reduce the chance for false negative results we restricted our analysis to test-negative ILI patients whose samples were taken no later than 4 days after disease onset. Because RT-PCR is highly specific (> = 99%), the probability for a false positive sample is very low.

The case-series method is applicable to routinely collected data. Although the unprotected period was short and the number of vaccinees among reported cases low, the method gave reasonable results. Estimates for weekly cohorts do not differ significantly from each other and from the estimate for the total season. Overall, VE estimates of the weekly age group cohorts are all above 50% and it is reassuring that they vary within a rather narrow corridor (Fig. 5). It has to be noted that we have not weighted the estimate of the VE for the total season because the size of the weekly cohorts was unknown (see Fig. S1 and Appendix S1). However, even a weighted estimate should be very similar because the weekly estimates lie within a close range. To further refine the methodology to calculate an overall estimate, approaches such as those used in meta analysis, may be considered [15].

Both methods were able to take into account that the epidemic wave evolved at the same time with the vaccination campaign. To do this, the test-negative case-control method added illness week into the multivariate model while the case-series method used the number of incident reported cases by week to calculate the proportion of expected vaccinated cases if the vaccine had no effect. A strength of both methods is that selection processes have limited effect because they apply equally to the entire study population.

Vaccination against seasonal influenza was not significantly associated with pIV infection. Several articles have published their analyses regarding the effect of seasonal trivalent influenza on the occurrence of pIV infection, but results were contradictory. A lack of effect [16], [17], [18], a preventive effect [19], [20] and even a “harmful” effect have all been reported [21]. With the power available in our dataset we would have detected even a small effect, for example if the OR for pIV would have been greater than 1.2 or smaller than 0.8.

Because the vaccine used in Germany was a vaccine adjuvanted by the squalen AS03 we cannot make statements about non-adjuvanted vaccines which may have different VE than the one used in Germany. When we attempted to compare our results with studies on the efficacy of squalen-adjuvanted **seasonal** influenza vaccines, we were unable to identify any in the published literature. However, clinical protection against seasonal influenza provided by inactivated, **non-adjuvanted** vaccines has been reviewed by Nichol who based her assessment on several published meta-analyses of mostly randomized controlled trials [22]. Depending on the results of different meta-analyses, effectiveness against laboratory-confirmed influenza in children ranged from 54–65% and in younger adults (aged younger than 65 years) from 63–80%. For elderly (aged more than 60 years) the only conducted randomized controlled trial found an efficacy of 58% [23]. With all due caution the effectiveness presented in this paper seems to be better for children and comparable in younger adults.

Our methods have several limitations. The case-series method is prone to influences biasing the case count during the unprotected period versus the protected period. This may be the case if physicians tend to sample vaccinated persons in the two weeks after vaccination more than in the protected period or if vaccinated persons get vaccinated when they become aware of influenza cases in their (private) surrounding, but are less eager to become vaccinated when the epidemic wave has passed and the risk has therefore diminished. Then the decision to become vaccinated and the risk for infection in the unprotected period may reflect to some degree not only the force of infection in the population as a whole but particularly in their immediate environment. However, the distribution of cases after vaccination (Fig. 3) does not suggest that these potential biases are of substantial magnitude.

On a similar note, we have not taken into account the dynamic geographical course of the epidemic which also may have affected vaccination and disease status. Here, two scenarios are possible: (i) individuals have been vaccinated when the epidemic was approaching (staggered vaccination), (ii) individuals have been vaccinated when the vaccine became available (vaccination largely at the same time). Some simple calculations simulating the first scenario showed small deviations of about 5% of the calculated vaccine effectiveness; however, in the scenario where most persons were vaccinated in the first weeks when the vaccine became available, i.e. vaccination was mainly triggered by availability of the vaccine, would not have resulted in a different effectiveness. As it turned out vaccination coverage remained low in Germany as only 8% of the population was vaccinated [24]. In particular, during the start of the campaign it was focused on priority groups, such as health care personnel, first responders, persons with chronic underlying diseases and pregnant women. Scenario two seems therefore more realistic. Another way to analyse this issue would have been to stratify by geographical region which would have necessitated a larger number of vaccinated cases than were available.

In the case series method it was necessary to use cut-off points for the end of the unprotected and the beginning of the protected period after vaccination which could be challenged. The work by Brokstad et al. showed an increase in antibody titers 8–9 days post vaccination [12]. After that an increase roughly following a saturation curve can be expected resulting in protective antibody titres in the majority of vaccinees about two weeks post vaccination [10]. Data from recent studies confirm these findings also for the pandemic vaccine [11], [25]. To explore the influence of different cut-off points on the VE estimates we had calculated the effect when the unprotected period was assumed to last until day 6,7,8,9 and 11 post vaccination. Lowering the unprotected period from 9 to 6 days increased the VE stepwise to roughly 3% at day 6, and dropped by 2% when the unprotected period was extended to 11 days. We concluded that the choice of the cut-off for the unprotected period is minor and that the results generated by the method are fairly robust. Nevertheless, a degree of imprecision remained because we used weekly values for the force of infection and the assumption that the cohort had been vaccinated by Wednesday.

Compared to our estimates, a recent publication using the screening method found a higher VE than presented here (97% for persons aged 14–59 years, 83% for persons 60 years and older) [26]. In general, the screening method may encounter difficulties when assessing VE during an ongoing vaccination campaign because the dynamic of the epidemic, the change of the proportion vaccinated in the population over time and the time that vaccinations need to take effect need to be considered. In the above mentioned paper, the authors tried to take these issues into account by beginning their study period three weeks after initiation of the vaccination campaign when estimated vaccination coverage in the population was already 4%, and coverage increased only by an additional 3% over the next 3 months [26]. In addition, selection processes like a lower likelihood to swab vaccinated persons or different laboratory sensitivity for different age groups in relation to the proportion vaccinated in these age groups may influence the estimated VE. A case control study from England and a European multicenter case control study showed similar estimates than our study (71% [27] and 72% [25](imputation) or 66% respectively). One study found indications for a lower protection in the elderly [25] and the other study indicated a higher protection in the younger age group [27], which is in line with the results of our study. Interestingly one study indicated a possible protection in the period 8 to 14 days after vaccination which potentially may challenge the assumption of a negligible protection in the first seven days after vaccination [25]. However the confidence intervals of the estimation are very wide. In our data we observed a very steep drop of cases 7 to 9 days after vaccination (Fig. 3) suggesting that immunity starts to take effect approximately one week after vaccination. A thorough comparison of all methods and findings (including other studies) may give more insight to explain differing results.

In conclusion both methods provided evidence for the good VE of the AS03-adjuvanted vaccine against the pandemic virus A/(H1N1) 2009. Should this virus remain the dominant virus or one of the viruses circulating in the human population, this or a similar vaccine should provide satisfactory protection against disease.

## Supporting Information

Appendix S1 **pooling the over all-VE based on the weekly cohorts.**
(DOC)

Figure S1 **Formula used for pooling the over all-VE based on the weekly cohorts: With:** - RR = relative risk - x ranges from 44 (the first week where vaccination started in an organized fashion) to 49 (where vaccination activity and the epidemic declined) - for all weeks to be considered there must be two or more cases in the unprotected and protected period in the cohort vaccinated in week x - y = interval in days chosen from vaccination until illness onset (unprotected period).
(DOCX)
