# Supplementary material for: Effectiveness of the AS03-Adjuvanted Vaccine against Pandemic Influenza Virus A/(H1N1) 2009 – A Comparison of Two Methods; Germany, 2009/10
Source: PLoS One. 2011 Jul 18;6(7):e19932. doi: 10.1371/journal.pone.0019932 (PMC3138735; doi:10.1371/journal.pone.0019932)
Supplement: Appendix S1 — pooling the over all-VE based on the weekly cohorts. (DOC) [file pone.0019932.s001.doc]

**Appendix S1**

Example of calculations and pooling of results, case-series method:

| 1 | 2 | 3 | 4 | 5 | 6 | 7 | 8 | 9 | 10 | 11 |
| --- | --- | --- | --- | --- | --- | --- | --- | --- | --- | --- |
| Week | Force of infection, week x, * 4/7 | Force of infection, week(x+1), * 5/7 | Force of infection, protected period | Cases among vaccinated in week x during unprotected period | Term 1  (col.4 / col.5) | Weeks of the unprotected period | Cumulative force of infection during protected period (week(x+2)-week(53)) | Cases among vaccinated in week(x) during protected period (week(x+2)-week(53)) | Term 2 (col.8 / col.9) | Term 1/  term 2 |
| week 44 | 30 | 40 | 70 | 7 | 10 | 46-53 | 60 | 2 | 30 | 0,33 |
| week 45 | 32 | 35 | 67 | 8 | 8,4 | 47-53 | 57 | 1 | 57 | 0,15 |
| Pooled | 62 | 75 | 137 | 15 | 9,1 |  | 117 | 3 | 39 | 0,23 |

Column 11 expresses the relative risk
