# Supplementary material for: Effectiveness of the AS03-Adjuvanted Vaccine against Pandemic Influenza Virus A/(H1N1) 2009 – A Comparison of Two Methods; Germany, 2009/10
Source: PLoS One. 2011 Jul 18;6(7):e19932. doi: 10.1371/journal.pone.0019932 (PMC3138735; doi:10.1371/journal.pone.0019932)
Supplement: Supplementary file 2 [file pone.0019932.s002.docx]

$$\frac{\sum_{x=44}^{49} \text{reported cases of week } x * 4/7 + \text{reported cases of week } (x+1) * 5/7}{\sum_{\text{[illegible]}}^{49} \text{reported cases during the unprotected period (illness onset within y days after vaccination) of the cohort vaccinated i[illegible]}}$$
